# A remarkable new pygmy grasshopper (Orthoptera, Tetrigidae) in Miocene amber from the Dominican Republic

**DOI:** 10.3897/zookeys.429.8020

**Published:** 2014-07-30

**Authors:** Sam W. Heads, M. Jared Thomas, Yinan Wang

**Affiliations:** 1 Illinois Natural History Survey, Prairie Research Institute, University of Illinois at Urbana-Champaign, 1816 South Oak Street, Champaign, Illinois 61820, USA; 2 1101 South Arlington Ridge Road, Arlington, Virginia 22202, USA

**Keywords:** Orthoptera, Tetrigidae, pygmy locust, grouse locust, Hispaniola, Caribbean, amber, fossil

## Abstract

A new genus and species of pygmy grasshopper (Orthoptera: Tetrigidae) is described from Early Miocene (Burdigalian) Dominican amber. *Electrotettix attenboroughi* Heads & Thomas, **gen. et sp. n.** is assigned to the subfamily Cladonotinae based on the deeply forked frontal costa, but is remarkable for the presence of tegmina and hind wings, hitherto unknown in this subfamily.

## Introduction

The Tetrigidae (pygmy grasshoppers, grouse locusts or ground hoppers) are a diverse group of small orthopterans characterized by their often remarkable morphological crypsis. These diminutive insects are primarily ground-dwelling and most commonly encountered among leaf litter on the forest floor, or in wet, marshy habitats bordering rivers, streams or standing water. With more than 1,700 species in over 250 genera, the Tetrigidae are, among Caelifera, second only to Acrididae in terms of species diversity (Steinmann 1962, 1969, 1970, 1971; Otte 1997). The family has a cosmopolitan distribution and is most diverse in the tropics (Hancock 1907; Rentz 1991; Heads 2009a). Tetrigids are herbivorous and feed primarily on bryophytes and algae, and occasionally on lichens and small vascular plants (Verdcourt 1947; Hodgson 1963; Reynolds et al. 1988; Hochkirch et al. 2000; Kočarek et al. 2008). Due to their affinity for wet and semi-aquatic habitats, many tetrigids are capable swimmers both above and beneath the water surface (Hancock 1902; Lucas 1920; Amédégnato and Devriese 2008). In spite of their diversity and fascinating life histories, the tetrigids remain one of the most neglected groups of Orthoptera and little is known of their biology or evolution (Hochkirch et al. 2006; Heads 2009a; Kočarek et al. 2011).

Members of the family are readily distinguished from other orthopterans by the marked posterior elongation of the pronotum which covers the entire dorsal surface of the abdomen and often extends well beyond it as an acuminate process (Hancock 1902, 1907; Rentz 1991; Heads 2009a). In addition to their comparatively small size, tetrigids also share several morphological characters with Tridactyloidea (in particular, Tridactylidae and Ripipterygidae), including the reduction of the pro- and mesotarsi to only two tarsomeres, the absence of arolia between the pretarsal claws, and the presence of a precoxal bridge connecting the pronotum to the prosternum (Rentz 1991; Heads 2009a, b, 2010). In both tetrigids and tridactylids/ripipterygids, the tegmina are markedly reduced in both size and venation, or absent entirely. When present and well-developed, the hind wings of both groups have M closely associated or fused with R, and all longitudinal veins unbranched except for a basal division of Cu (Heads 2009a, 2010). These similarities have long been considered as supporting a close relationship between Tetrigidae and Tridactyloidea, and the two have traditionally been united at either superfamilial or infraordinal rank (e.g. Beier 1955; Dirsh 1961; Sharov 1968). However, such a relationship is not supported by molecular analyses which have consistently failed to recover a tetrigid-tridactyloid clade, instead resolving Tetrigidae as sister to Acridomorpha to the exclusion of Tridactyloidea (e.g. Rowell and Flook 1998; Flook et al. 1999).

Tetrigidae are extraordinarily rare in the fossil record. To date, only nine species have been described (Table 1) of which, five (including the new fossil described herein) are from fossil resins. The oldest fossil tetrigids are known from compression fossils in Early Cretaceous vulcano-sedimentary deposits of the Turga Formation, Transbaikalia, Russia. Sharov (1968) described two monotypic genera from this deposit, namely *Archaeotetrix locustopseiformis* and *Prototetrix reductus*, known from partially preserved body fossils and isolated wings. Both *Archaeotetrix* and *Prototetrix* possessed well-developed tegmina and wings with primitive venation (Sharov 1968, figs 38a–c and pl. viii) and likely represent stem-Tetrigidae. Two tetrigids are known from Eocene Baltic amber: *Acrydium bachofeni* Zeuner, 1937 and *Succinotettix chopardi* Piton, 1938. Gorochov (2010) speculated that *Acrydium bachofeni* may be a second species of *Succinotettix* or even conspecific with *Succinotettix chopardi*, though he did not examine the types of either species. Piton’s holotype of *Succinotettix chopardi* is probably lost but Zeuner’s holotype of *Acrydium bachofeni* was recently located in the Bachofen-Echt collection at the Bayerische Staatssammlung für Paläontologie und Geologie in Munich, Germany (M. Nose pers. comm. 2014) and will be redescribed in a future paper. The only other Eocene representatives of the family are organic compression fossils from lacustrine deposits of the Green River Formation and were recently described and named *Eotetrix unicornis* by Gorochov and Labandeira (2012). The latter authors did not publish photographs of their specimens and it would appear that they are rather poorly preserved, thus limiting interpretation. However, it is clear from their line drawings that *Eotetrix unicornis* bears some resemblance to certain Neotropical Batrachideinae and is strikingly similar to the extant genus *Scaria* Bolívar, 1887 in the form of the anterior pronotal process. Miocene tetrigids are known as compression fossils from the Late Miocene of Oeningen, Switzerland (*Tettigidea gracilis* Heer, 1865) and as inclusions in Early Miocene amber from the Dominican Republic (*Antillotettix electrum* Heads, 2009a and *Baeotettix lottiae* Heads, 2009a).

Here, we describe a new genus and species of tetrigid from Dominican amber. Like other Dominican amber Tetrigidae, the new genus belongs to the subfamily Cladonotinae; a circumtropical group defined primarily by a deeply forked frontal costa (Hancock 1902, 1907; Pérez-Gelabert et al. 1998; Heads 2009a). Unlike other members of the subfamily however, the new genus possesses both tegmina and hind wings, which are absent in all other cladonotines.

**Table 1.** Alphabetized list of fossil Tetrigidae described to date with their geological and geographical provenance. Asterisks indicate that the whereabouts of the type specimen is unknown.

Species	Locality and age
*Acrydium bachofeni* Zeuner, 1937	Baltic amber (Middle Eocene)
*Antillotettix electrum* Heads, 2009	Dominican amber (Early Miocene)
*Archaeotetrix locustopseiformis* Sharov, 1968	Turga Fm, Transbaikalia, Russia (Early Cretaceous)
*Baeotettix lottiae* Heads, 2009	Dominican amber (Early Miocene)
*Electrotettix attenboroughi* Heads & Thomas, this paper	Dominican amber (Early Miocene)
*Eotetrix unicornis* Gorochov, 2012	Green River Fm, Wyoming, USA (Middle Eocene)
*Prototetrix reductus* Sharov, 1968	Turga Fm, Transbaikalia, Russia (Early Cretaceous)
*Succinotettix chopardi* Piton, 1938*	Baltic amber (Middle Eocene)
*Tettigidea gracilis* Heer, 1865*	Oeningen, Switzerland (Late Miocene)

## Material and methods

The holotype is deposited in the Paleontology Collection of the Illinois Natural History Survey (INHS), at the University of Illinois. The piece of amber contained multiple insect and plant inclusions and was cut into three pieces in order to better view the specimen. Cuts were made using a jeweler’s saw with care taken not to damage other inclusions. Facets were then ground flat and given a final polish with 50,000 mesh diamond paste to remove visible scratches. The holotype of *Electrotettix attenboroughi* was studied using Olympus SZX12 zoom stereomicroscope with 1× and 2× objectives and a Zeiss SteREO Discovery V.20 stereomicroscope with 0.63× and 1.5× objectives. Photomicrographs were produced using an AxioCam HRc Rev. 3 digital camera attached to the Zeiss. Images were focus-stacked using Helicon Focus version 5.3 and the panorama was stitched in Adobe Photoshop CS5. Illustrations were produced using Adobe Illustrator CS5. The age and origin of Dominican amber is reviewed by Iturralde-Vinent and MacPhee (1996), Iturralde-Vinent (2001), Grimaldi and Engel (2005) and Penney (2010). Terminology follows that of Heads (2009a).

## Systematic palaeontology

### Order Orthoptera Olivier, 1789
Suborder Caelifera Ander, 1936
Family Tetrigidae Audinet-Serville, 1838
Subfamily Cladonotinae Bolívar, 1887

#### Genus Electrotettix

Heads & Thomas gen. n.

http://zoobank.org/FBFDB93E-802F-4639-8063-86AFBB4D83A5

##### Type species.

*Electrotettix attenboroughi* Heads & Thomas, gen. et sp. n.

##### Diagnosis.

The new genus is distinguished from all other Cladonotinae by the presence of tegmina and vestigial hind wings. Frontal costa forked just superior of antennal torulae. Anterior margin of frons at frontoclypeal margin broadly emarginate. Antennal flagellomere 3 approximately half as long as other flagellomeres. Pronotum with distinct lobe superior to humeral sinus. Posterior margin of pronotum forming a slightly upturned, blunt acuminate process.

##### Etymology.

The genus-group name is a combination of *electrum* (Latin from Greek, meaning “amber”) and *tettix* (Greek, meaning “grasshopper”).

#### Electrotettix attenboroughi

Heads & Thomas sp. n.

http://zoobank.org/C2884326-0785-414A-B9AF-243A52B53F82

**Figures 1–2.**
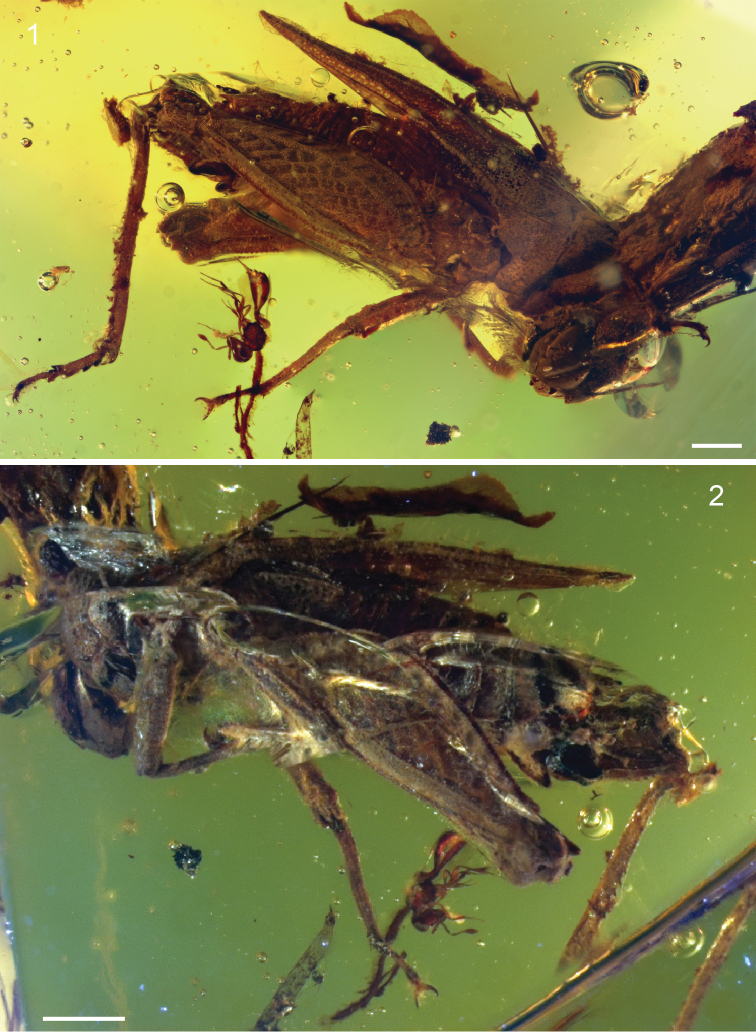
*Electrotettix attenboroughi* Heads & Thomas, gen. et sp. n. **1** holotype in oblique right lateral view (scale bar 1.0 mm) **2** holotype in oblique left lateral view (scale bar 1.0 mm).

Figs 1 [Fig F2] [Fig F3] [Fig F4] –7

##### Diagnosis.

As for the genus (see above), by monotypy.

##### Description.

**Figure 3. F2:**
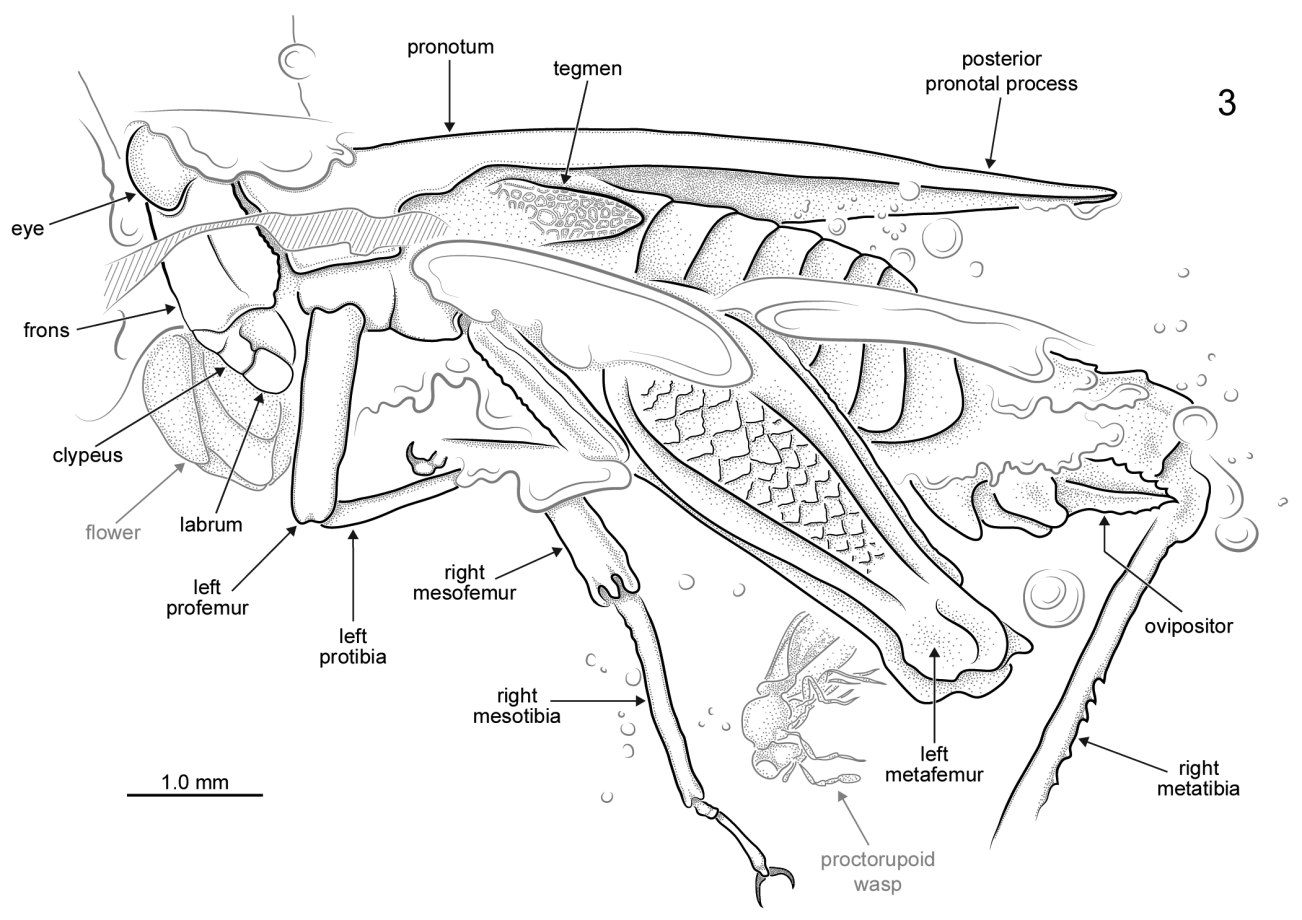
*Electrotettix attenboroughi* Heads & Thomas, gen. et sp. n., explanatory drawing of holotype in oblique left lateral view (scale bar 1.0 mm).

**Figure 4. F3:**
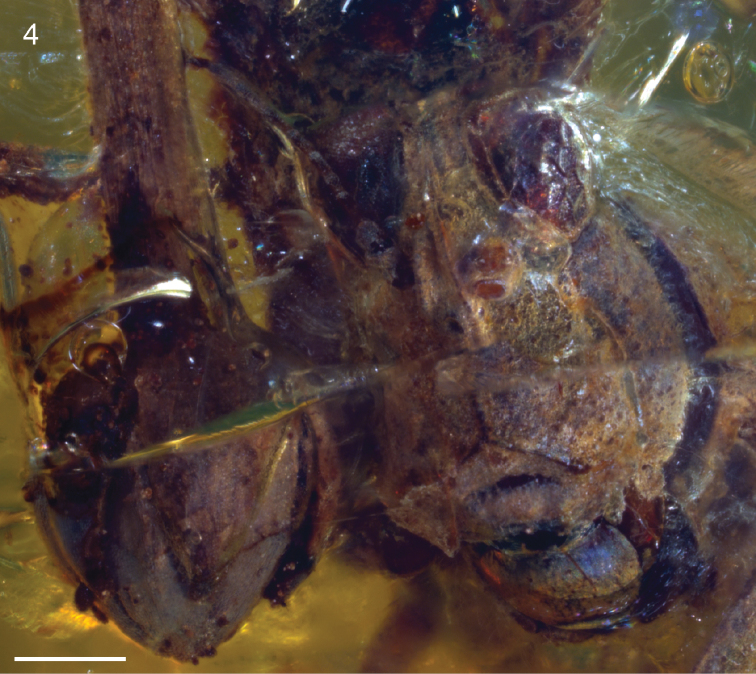
*Electrotettix attenboroughi* Heads & Thomas, gen. et sp. n., frontal view of head capsule; the flower bud preserved alongside the head is visible to the left (scale bar 0.5 mm).

**Figure 5. F4:**
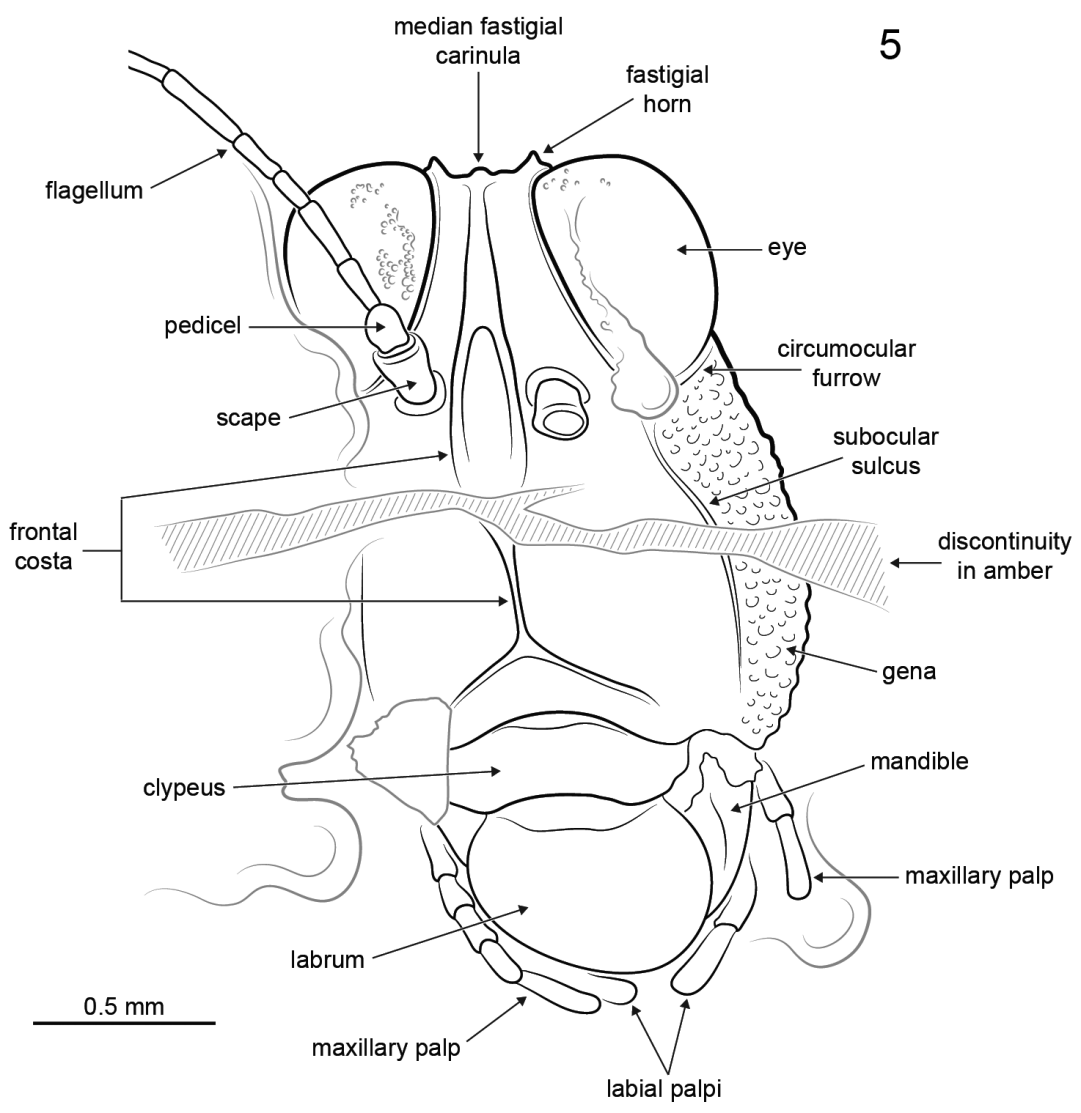
*Electrotettix attenboroughi* Heads & Thomas, gen. et sp. n., explanatory drawing of head capsule in frontal view (scale bar 0.5 mm).

*Female*: Approximately 8.0 mm long measured from fastigium verticis to posterior apex of pronotum (Figs 1–3). Head hypognathous, robust and dorsoventrally elongate (Figs 4–5). Integument granulose; genae markedly so, bearing numerous tuberculae. Compound eyes large, globose, projecting somewhat dorsally; ventral margin acutely rounded. Vertex with low median carinula becoming lower as it crosses the fastigium, and two stronger, well-defined lateral carinae forming small dorsolaterally produced fastigial horns between compound eyes. Lateral foveae deep, longer than wide, deeper anteriorly than posteriorly. Fastigium verticis not projecting anteriorly beyond compound eyes. Interocular distance *c.* 0.30 mm. Frontal costa nascent immediately beneath fastigium, becoming prominent *c.* 0.25 mm from fastigial ridge and bifurcating at the lateral ocelli, diverging into two prominent ridge-like costal lobes between antennal torulae and ending at median ocellus. Frontal carina bifurcating *c.* 0.52 mm beneath median ocellus. Fronto-clypeal margin distinct, broadly emarginate Clypeus narrow; anterior margin with broad, shallow emargination. Labrum shield-like, markedly larger than clypeus with rounded apex. Mandible robust. Gena somewhat inflated with strongly granulose/tuberculate ornament; delimited anteriorly by a deep subocular furrow running the entire length of the fronto-genal region. Antennae filiform, with at least ten flagellomeres. Scape subcylindrical, approximately twice as large as pedicel and somewhat compressed laterally. Pedicel subspherical, narrower than scape but wider than flagellomeres. Flagellum at least 1.24 mm long. Flagellomeres cylindrical, longer than wide; flagellomere 3 approximately half as long as the others.

**Figures 6–7.**
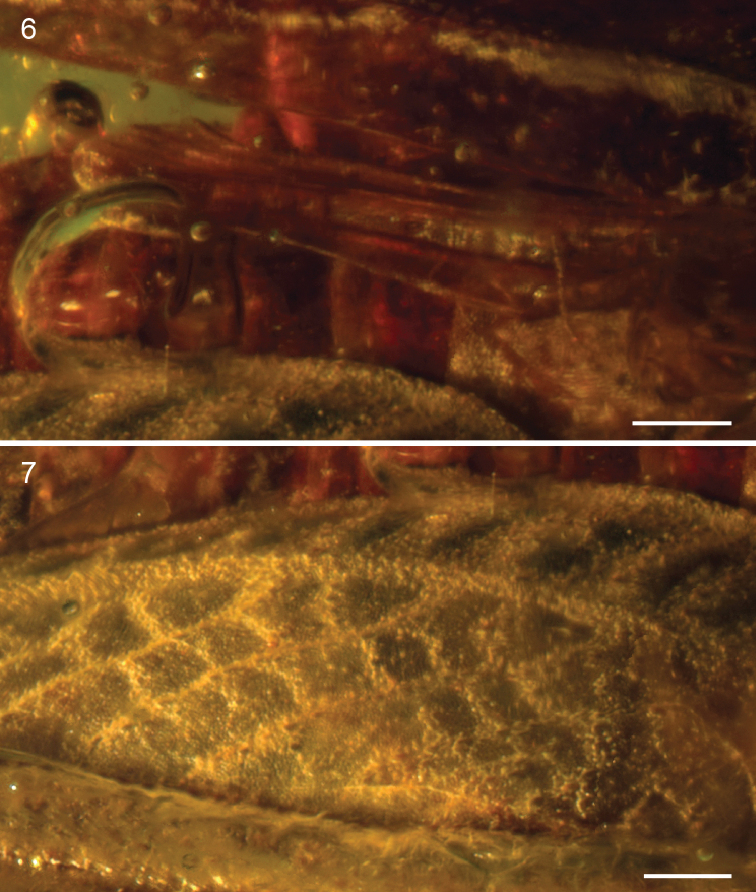
*Electrotettix attenboroughi* Heads & Thomas, gen. et sp. n. **6** right hind wing (scale bar 0.25 mm) **7** detail of the superior marginal and medial areas of right metafemur (scale bar 0.25 mm).

Pronotum robust, *c.* 6.55 mm long, with coarsely granulose ornament; anterior margin with small tectate process extending slightly above vertex of head; posterior process almost reaching apex of abdomen and terminating in a blunt and slightly upturned acuminate tip. Median carina forming distinct keel. Lateral carinae well-developed. Transverse sulci distinct, crossing and cutting the lateral carinae but not cutting the median carina. Thoracic sterna robust. Tegmen present, scale-like, longer than wide; venation indistinct, comprising numerous closed cells. Hind wing approx. 2.5 mm long, tightly folded (Fig. 6); costal lobe well-developed; Sc reaching costal margin almost reaching apex of wing; R and M entirely fused, running very close to Sc; area between R+M and Cu with numerous crossveins; CuA not visible; CuP approximating running close to 1A for its entire length; anal veins numerous.

Profemur 1.75 mm long, subquadrate in section with poorly developed carinae. Protibia at least 1.5 mm long and markedly more slender than profemur. Protarsus largely obscured by bubbles and debris in the amber. Mesofemur quadrate in section and similar in length to profemur but with carinae well-developed and complete for entire length of femur. Mesotibia 1.5 mm long, more slender than mesofemur but not as slender as protibia. Mesobasitarsus 0.2 mm long with bilobed euplantulae; second tarsomere 0.7 mm long, apically inflated with two strong pretarsal claws. Metafemur large and robust (Fig. 7), 5.5 mm long, with prominent upper and lower carinulae, dorsal keel and ventral carinae; superior and inferior marginal areas with transverse patches of rugose integument separated by smooth cuticle; medial area with prominent herringbone ornamentation comprising roughly diamond-shaped ‘cells’ of smooth cuticle, delimited by raised areas of rugose integument; femoral lobe low and distinctly rounded, not forming a spine; genicula bulbous with prominent dorsal process. Metatibia 5.0 mm long with prominent genicular bulb; 6 inner and 6 outer robust dorsal spines; 2 inner and 2 outer curved apical spurs, with inner spurs longer than outer spurs. Metabasitarsus robust, 1.0 mm long, with distinct, dorsal apical spine and two bilobed euplantulae situated in its basal half; second tarsomere much shorter, 0.3 mm long; third tarsomere almost as long as basitarsus, distinctly curved and somewhat inflated apically, bearing two pretarsal claws.

Abdomen at least 4.0 mm long, though apical damage prevents accurate measurement. Subgenital apically bilobed. Ovipositor approximately 1.0 mm long, with strong denticles on dorsal valvulae and fewer, smaller denticles on the ventral valvulae. Dorsal parts of terminalia (epiproct, cerci, etc.) obscured by bubbles and detritus.

*Male*: Unknown.

##### Holotype.

INHS 10175, Early Miocene (Burdigalian) amber from the La Toca region, near Santiago de los Caballeros, Santiago Province, Dominican Republic. Well-preserved adult female in a piece of amber approximately 20 × 15 × 12 mm. Numerous syninclusions are also present within the piece, including: an indet. chalcid wasp (Hymenoptera: Chalcidoidea); an indet. proctotrupoid wasp (Hymenoptera: Proctotrupoidea); a single worker ant of the genus *Solenopsis* (Hymenoptera: Formicidae); numerous smaller ants, possibly of the genus *Azteca* (Hymenoptera: Formicidae); a springtail (Collembola); three net-winged midges (Diptera: Blephariceridae); numerous botanicals including a well-preserved flower bud and a leaf fragment with possible epiphytic fungus.

##### Etymology.

The specific epithet is a patronym honouring Sir David Attenborough, British naturalist and film maker, who has been an inspiration not only to the authors of this paper, but to an entire generation of natural scientists.

##### Remarks.

*Electrotettix* is very distinctive among Neotropical cladonotines in that it possesses tegmina and rudimentary hind wings. Although the hind wings are much reduced, they nevertheless have complete venation and demonstrate full rotation, confirming that the holotype is a brachypterous adult and not a nymph with wing pads. *Electrotettix* is similar to *Baeotettix* in the large eyes projecting dorsally above the fastigium, but differs in the smaller fastigial horns and the absence of superior lobes on the frontal costa. Both *Baeotettix* and *Electrotettix* share features in common with extant Antillean cladonotines such as *Bahorucotettix*, *Haitianotettix*, *Mucrotettix* and *Hottotettix* (Pérez-Gelabert et al. 1998) including the densely rugose integument, compact body form and presence of fastigial horns, though the latter are not present in all Antillean cladonotines, being absent from genera such as *Truncotettix* and *Antillotettix* (Pérez-Gelabert 2003).

## Discussion

Fossil taxa are widely recognized as a valuable source of data concerning the morphology and evolution of their extant relatives. Such taxa often present novel combinations of plesiomorphic and derived character states, which provide unique insight into the acquisition and transformation of morphological characters through deep time (Donoghue 2005; Grimaldi and Engel 2005; Heads 2008). The presence of wings, however well-developed, among fossil representatives of exclusively wingless modern taxa is often an indicator of their basal position with respect to the crown group, as is thought to be the case for the fossil proscopiid grasshopper, *Eoproscopia* from the Cretaceous of Brazil (Heads 2008). However, care must be taken when interpreting such occurrences in taxa that are otherwise highly derived. *Electrotettix* is clearly related to a group of Antillean cladonotines characterized by a coarsely granulose integument and comparatively low pronotal crest (Pérez-Gelabert et al. 1998; Pérez-Gelabert 2003), but remarkable for the presence of tegmina and hind wings which are unknown in any other Cladonotinae, Antillean or otherwise (Hancock 1907; Günther 1938; Blackith 1992; Heads 2009a). While the presence of wings may be considered plesiomorphic, it is unlikely that *Electrotettix* is basal to all extant cladonotines given that it shares several characters with modern Antillean genera (see remarks above). If a close relationship between *Electrotettix* and extant Antillean taxa is confirmed, it would suggest that wings were lost at least twice within the subfamily.

While a robust comparative phylogenetic analysis is not yet available for Cladonotinae, there is evidence to support the existence of an Antillean clade characterized by coarsely granulose integument, presence of fastigial horns or tubercles, and low, non-foliaceous pronotal crests. Such a clade would comprise the fossil genera *Baeotettix* and *Electrotettix* as well as all extant Antillean cladonotines with the exception of the leaf-mimics *Choriphyllum* and *Phyllotettix* (see Heads 2009a). However, with a detailed phylogenetic analysis lacking, the precise relationships of the cladonotine genera (and indeed all Tetrigidae) remain uncertain and a great deal more work is needed before such questions can be adequately addressed. Nevertheless, it is clear from the great diversity of Antillean cladonotines, that the group have undergone rapid diversification in the Caribbean region. Of the thirty or so tetrigid species now known from the West Indies, over 70% are cladonotines (Heads 2009a). This diversity contrasts markedly with that of mainland South and Central America, where the tetrigid fauna is dominated by Batrachideinae, Lophotettiginae and Tetriginae (Heads 2009a). Pérez-Gelabert et al. (1998) and Heads (2009a) postulated that the diversification of Cladonotinae in the Caribbean was a result of radiations fueled by frequent vicariance events resulting from the dynamic geological evolution of the Antillean archipelago. Cladonotinae are unable to fly and are therefore, more sensitive to geographic isolation than other taxa. While *Electrotettix* did possess wings, they were much reduced and would not have allowed the animal to fly. While an understanding of the evolution of Caribbean biota is difficult given the complex geological history of the region (Grimaldi and Engel 2005), the limited dispersal potential of cladonotines would almost certainly have contributed to their diversification among the many isolated islands, mountain ranges and valleys of the Antilles.

## Supplementary Material

XML Treatment for Electrotettix

XML Treatment for Electrotettix attenboroughi
